# Applying a Simple Model of Cost Effectiveness Study of HPV Vaccine for Iran

**Published:** 2015

**Authors:** Mohsen Khatibi, Hamid Reza Rasekh

**Affiliations:** Department of Pharmacoeconomics and Pharmaceutical Administration, School of Pharmacy, Shahid Beheshti University of Medical Sciences, Tehran, Iran.

**Keywords:** Cost effectiveness, Simple model, HPV vaccine, Iran

## Abstract

HPV vaccine has been recently added to the Iran Drug List, so decision makers need information beyond that available from RCTs to recommend funding for this vaccination. Modeling and economic studies have addressed some of those information needs. We reviewed cost effectiveness studies to find a suitable model for Iranian population to determine the potential cost effectiveness of HPV vaccine program based on domestic available epidemiologic data. Articles were obtained from an extensive literature search to determine the cost effectiveness of implementing an HPV vaccination program with routine cervical cancer screening. A total of 64 studies were included in this review. Although the studies used different model structures, baseline parameters and assumptions (either a Markov, Hybrid, or Dynamic model). Most of the proposed cost effectiveness models need to model the probability of HPV acquisition, the possible progression from HPV infection to CIN I, CIN II, CIN III and cervical cancer, the probability of HPV transmission which are not available in Iranian epidemiologic data. Based on the available epidemiologic data in Iran, the simplified and it requires substantially fewer assumptions than the other more complex Markov and hybrid models, therefore we decided to use this model for the evaluation of cost effectiveness of HPV vaccine in Iran.

## Introduction

The human papilloma virus (HPV) is among the most common sexually transmitted viruses. Chronic infection with certain subtypes of the HPV is the primary cause of cervical cancer and its precancerous lesions. At least 50% of the adult population is infected with this virus during their lifetime. Despite screening programs for cervical cancer, it remains the second most common cause of cancer-related death among women worldwide (1,2).

Gardasil is a quadrivalent vaccine of subtypes 6, 11, 16 and 18 of the HPV. On the average, 70% of cervical cancers are caused by infection with subtypes 16 and 18, and 90% of genital wards are caused by subtypes 6 and 11 of the HPV (3,4). Vaccines are essential tools for preventing the diseases. They will protect the vaccinated individual and help to protect the community by reducing the spread of infectious agents (5). There is no completely secure way for protecting sexually active adults against genital warts, and the current therapy modalities are often painful, time-consuming and with high risk of recurrence. Therefore, Gardasil vaccine may be quite helpful with its protective properties. Gardasil is administered for women aged 9-26 years for preventing diseases caused by the HPV subtypes 6, 11, 16 and 18, including cervical cancer, genital warts, and precancerous or dysplastic lesions (6).

Gardasil vaccine is part of the national immunization program in the United States, Canada and Australia, among others. It is covered by insurance in Canada and Australia. Gardasil has been registered and is in use in 124 countries. Moreover, it is part of the national immunization program in 19 countries of the world which means it is administered to all girls aged 11-12 years (7).

In June 2006, the American Food and Drug Administration (FDA) approved Gardasil vaccine (produced by Merck & Co.) for girls aged 9-26 years (8).

The efficiency of Gardasil is 100% if administered prior to the first sexual contact (8). Numerous studies have been conducted to evaluate its cost-effectiveness by calculating the cost necessary for one quality-adjusted life year. None of them were cost saving for quadrivalent HPV vaccine (9). These include various models such as Markov model, decision model, dynamic model, transmission or a combination (9). 

Studies conducted so far have mostly addressed its impact on cervical cancer and cervical intraepithelial neoplasia (CIN) (10). Few studies have dealt with the cost-effectiveness of Gardasil regarding other malignancies caused by the HPV (11).

The national immunization program of girls prior to sexual activity has been shown to reduce HPV-related mortality and morbidity and to be cost-effective (12-16). Since HPV infection is asymptomatic, it is growing silently (17). This vaccine is also recommended for 13-26-year-old females even if the female is already sexually active and could have contracted HPV infection (8). 

Vaccination can cause immunity both directly and indirectly through herd immunity (18). For the HPV vaccine, the exact total duration of protection is not known yet, because the current maximum length of clinical trials is around 6 years. Consequently, it could be argued that base-case analysis on the cost-effectiveness of the HPV vaccine should not use durations of protection beyond 6 years, let alone lifelong protection (19)

In order to determine the long term benefit of this vaccine and impact of vaccine program on the future rate of cervical cancer, many pharmacoeconomists used mathematical model. Some models focused on cost effectiveness of different strategies (20-23). 

 Our objective is to review these cost effectiveness studies to find a suitable model for Iranian population to determine the potential cost effectiveness of HPV vaccine (Gardasil) program based on domestic available epidemiologic data. 

## Experimental

*Methods of literature review*

*Search strategy development*

In order to have a complete review of all cost-effectiveness models for HPV vaccine, we developed a search strategy. In this step “the content-related keywords” were defined and combined it with “AND” or “OR”. To assure the quality of the review and increasing the search sensitivity, we didn’t use “AND” frequently. 

As many diseases are related to HPV, in our search query we mentioned the following keywords based on PICO model: Papillomavirus, HPV and Human Papilloma Virus.

For searching the intervention part “vaccine”, “prevention” and “prophylactic” combined with “OR”.

The C-CERG strategy was used to search the title, abstract and keyword fields within records of both cost-effectiveness Studies and HTA reports. This method is the most documented search strategy in this field (24).

*Search in electronic database for economic evidences*

The search query was” (“human papilloma virus" OR papillomavirus OR HPV OR (papilloma AND viru*)) AND (vaccin* OR preven*)” without any limitations. NHSEED, HTA, DARE, CEA Registry, PEDE, Econlit and EURONHEED were our search databases. 

*Inclusion criteria and quality assurance*

All published English-Language studies were included in the review that assessed the ICER of HPV vaccine compared with other alternative strategy like cervical cancer screening. To find directly related studies, all obtained studies were categorized based on below criteria:

- *Directly* *relevant (R1):* containing the full economic evaluation studies, considering the cost of quadrivalent HPV vaccine as a main intervention compared to other alternatives.
- *Indirectly Relevant (R2):* Quadrivalent HPV vaccine was not evaluated as main intervention, using different target group (different age or gender), focused on diagnosis, treatment of cervical cancer or screening only, and other vaccines as a main alternative.
- *Irrelevant (R3):* Focus on other viral infection.

## Results

Through literature review, 39 studies in NHSEED database, 26 studies in HTA, and 10 studies in DARE were found. These three databases are related to CRD. Moreover, 46 studies in CEA Registry, 35 studies in PEDE, 2 studies in Econlit, and 26 studies in EURONHEED were addressed during the review of the literature. 

Overall, a total number of 241 studies were found of which, only 148 remained in second step after omitting duplicate studies among databases. For the next step and based on the inclusion criteria and relevancy criteria, 64 studies were categorized in R1, 81 studies in R2, and 3 studies in R3. After this step we had three expert panels to find the best and suitable model for Iran. 

In our study we defined an expert panel consists of 6 members; two gynecologists, two onco-gynecologists, one expert of systematic review and one expert of pharmacoeconomy. In the first expert panel and after reviewing 64 studies, 20 studies were defined as exactly related, which used defined models for their studies (Table 1). The result and summary of these studies categorized based on the used technologies, kind of economic study, effectiveness and cost data, kind of model, time horizon, and discount rate. After summarizing 20 studies, the second expert panel discussed the methods and needed data of the selected studies. Based on the feedback of this panel, 6 studies were selected for modeling which is appraised and summarized in the discussion section. Finally, in third expert panel and based on the available epidemiologic data in Iran and experts’ opinion, we defined one of these models as a basic model for Iranian cohort model. 

## Discussion

In this section, five major articles including Elbasha *et al*., 2007, Brisson *et al*., 2007, Kulasingam *et al.,* 2007, Bergeron *et al*., 2008, Kulasingam *et al.,* 2008 and Chesson *et al*., 2008 have been addressed in a thematic order as follows:

*Choice of Interventions or alternative interventions*

*Elbasha* *et al.,* *2007:*

The rationale for choosing alternatives is clear and precise in terms of addressing the current status of care (no vaccination strategy) alongside all possible vaccination options. Different conditions must be considered when applying the results. 

*Brisson* *et al.,* *2007:*

This study compares vaccination of young girls with anti HPV 16/18 and anti HPV 6/11/16/18 versus no vaccination plan. The latter represents the “do nothing” option, which is the current practice in the study site (Canada). Evidently, the status quo of service provision in the country must be assessed before trying to universalize the results.

*Kulasingam* *et al.,* *2007 (Australia):*

The rationale for selecting alternative interventions is clear and appropriate. The new approach to population-based immunization has been compared to the current standard practice in Australia. Nevertheless, it must be noted that the base strategy of cervical cancer screening alone may not be a suitable representative of the routine care practice in countries, which have already begun HPV vaccination. In sensitivity analysis, different vaccination programs (in different populations) have been considered, which may be logical and acceptable for other countries.

*Bergeron* *et al.,* *2008:*

Both interventions are reported clearly and elaborately enough. In addition, the choice of alternative strategy is very well justified. This strategy consists of screening from 25 to 65 years of age every three years in France.

*Kulasingam* *et al.,* *2008 (UK):*

Two options selected for prevention of cervical cancer are completely described. The profile of the population study, vaccination program and screening tests are mentioned.

*Chesson* *et al.,* *2008:*

No vaccination scenario as the second strategy was appropriate. 

*Validity of effectiveness and Benefit index estimation*

*Elbasha* *et al.,* *2007:*

Parameters of effectiveness have been adopted from published studies. However, the authors do not mention their search strategy or inclusion criteria. Also, the reason for selecting these particular estimates is not mentioned. The study mentions a review of literature but fails to indicate its strategies and methodology for review. Also, the information of the initial studies is not mentioned, making it impossible to assess validity of data from the initial studies. QALY estimation uses a decision-making tree model. The methods used for estimating desirability weights are not mentioned and are simply said to have been derived from published studies. Interest has been conducted appropriately. QALY is a good choice since it considers the most important health aspects (survival and quality of life) and provides a basis for comparison with other healthcare interventions.

*Brisson* *et al.,* *2007:*

Model parameters are derived from published studies. However, the authors do not mention the search strategies or inclusion criteria for selecting the initial studies. In addition, the study design is not specified. In general, it is difficult to evaluate the quality of effectiveness data in these studies. Using QALY as an index of benefits makes it possible to compare the findings of this study with others addressing vaccination programs and other interventions. The desirability coefficients for adjusted life expectancy based on quality of life are derived from published literature, but the study does not mention the method used for evaluating different health states. The interest rate of health benefits in the future is appropriate.

*Kulasingam* *et al.,* *2007 (Australia):*

Clinical data, for the most part, adopted from published studies, which are not mentioned, except in the case of data derived from the national database. Therefore, it is impossible to evaluate the validity of these estimations objectively without information regarding the scope, sample size, and follow-up procedures of the original studies, which served as source. However, extensive sensitivity analysis and choice of the most acceptable analysis value improve the power of clinical estimations. Using two benefit indices, with expected QALY values smaller than LY values, suggests the importance of evaluating quality of life in women with cancer. 

*Bergeron* *et al.,* *2008:*

The authors do not mention using a systematic review of the literature for finding all relevant effectiveness and clinical data. An explanation on the method of integrating and summarizing data obtained from studies has not been provided. Nevertheless, a summary about all parameters used in the model and their sources has been mentioned in the study. In addition, sources of desirability estimations are clearly mentioned.

*Kulasingam* *et al.,* *2008 (UK):*

Effectiveness data are obtained from a spectrum of published studies. However, the selection strategy is not mentioned. Clinical outcomes used for evaluating the advantages of two preventive strategies were selected in favor of vaccination and screening strategy. Some health benefits were excluded from the study. Desirability coefficients were adopted from a published and an unpublished study under supervision of experts and authors, which may cause some degree of bias. The reported data do not allow for evaluating methods of desirability assessment. The model structure is not presented visually. Nevertheless, a comprehensive description of different health states and possible transmissions has been provided.

*Chesson* *et al.,* *2008:*

The databases were relevant and valid. The treatment effects were based on trials, which characterized by high internal validity. The clinical and the utility valuations derived from the literature. The use of QALYs was appropriate because they capture the impact of the disease on patients’ health.

*Validity of cost estimation*

*Elbasha* *et al.,* *2007*:

It appears that cost analysis is performed from the payer’s point of view. All cost groups have been included in the analysis. Different cost groups are reported, although details of costs are not mentioned. The authors maintain that including indirect costs would reduce the desirability cost and thus improve the appeal of vaccination strategies. No specific source has been provided for this information. Mentioning the reference year of reported costs makes it easy to convert the costs for different time periods. Costs have not been statistically analyzed, but the changes in estimation of major costs have been included in sensitivity analysis.

*Brisson* *et al.,* *2007:*

Economical analysis is performed from the payer’s point of view, and all major cost items seem to have been included in the analysis. Uncertainty of cost data and consumed resources are addressed in sensitivity analysis. Future costs are interested appropriately. These factors improve the applicability of the findings. Moreover, the reference year of cost estimations are mentioned clearly, which makes it easier for future calculations.

*Kulasingam* *et al.,* *2007 (Australia):*

The cost groups considered in the study appear to be appropriate for the approach taken to analysis. Details of cost items are not given and some expenses are mentioned generally. Costs are obtained from national health care services, which reflect the local accounting systems. Consumed resources are obtained from published studies. Key assumptions of the study are addressed in sensitivity analysis. 

*Bergeron* *et al.,* *2008*:

The economical viewpoints used are clearly expressed. It seems that all cost items are considered based on their relation to the two viewpoints adopted. Sources of cost data (mainly from official French sources or articles published in France) are well presented. In addition, the authors have appropriately reported the time period of the study, interest rate, reference year of prices and currencies.

*Kulasingam* *et al.,* *2008 (UK):*

The costs considered in the model are an appropriate reflection of the viewpoint adopted (NHS). Methods of cost-assessment, modifications, sources of cost data and cost-service unit are presented appropriately and elaborately. Costs are modified for inflation rate. Nevertheless, the cost results of each strategy are not reported. Moreover, cost information is not mentioned for values consumed from each source.

Chesson *et al*., 2008:

The perspective was societal. The analysis of costs followed a similar approach to the clinical analysis, in that macro-categories were presented without a detailed breakdown of items. The cost estimates varied in the sensitivity analysis.

*Analysis and findings*

Elbasha *et al.,* 2007:

The authors state that their findings generally agree with those of previous studies. Nevertheless, the study yields considerable discrepancies with findings of other economic evaluations, which the authors attempt to account for. The study deals briefly with the issue of applicability of its findings in the section of sensitivity analysis. Alternative scenarios are considered in this section. The authors have also highlighted some strengths of their analysis, including use of reliable data, clarity and flexibility. Also, certain limitations of the study have been mentioned, including the fact that the model deals mainly with HPV transmission from the opposite sex. Nevertheless, many assumptions of the study are biased towards different vaccination strategies.

*Brisson* *et al.,* *2007:*

The authors do not seem to be biased in presenting their findings. Furthermore, the conclusion is a good reflection of the scope of analysis. The authors compare their findings with those from other countries and to some extent have managed to justify the discrepancies in desirability cost ratios.

*Kulasingam* *et al.,* *2007 (Australia):*

Cost and benefits are appropriately integrated. However, the overall sum of costs and benefits are only presented graphically and only the cost-effectiveness ratios are mentioned. Sensitivity analysis has been conducted and reported appropriately. A wide range of possible scenarios and alternative assumptions are addressed in sensitivity analysis, which indicates the power of the study.

*Bergeron* *et al.,* *2008:*

Details of the Markov model, which was used for modeling costs and outcomes of each intervention, are presented, but relevant diagrams are lacking in the text. The model was previously designed for the United States and then modified for the European status. Although a series of univariate sensitivity analyses were included to measure uncertainty of model findings, using a probabilistic sensitivity analysis may have provided a more comprehensive understanding of the model’s overall uncertainty. Methods and results are sufficiently explained. The limitations of the study are mentioned in the discussion section.

*Kulasingam* *et al.,* *2008 (UK):*

Crude costs and health outcomes are integrated as cost-effectiveness ratios. Observational epidemiologic data in England confirm the validity of parameters related to cervical cancer risk. Univariate sensitivity analyses are comprehensive and address all key parameters in an acceptable spectrum. While accepting the limitations of the study, the authors have attempted to justify them. These include the lack of powerful data on desirability coefficients of the health states in questions, lack of a probabilistic sensitivity analysis, and possibility of underestimating health benefits. The authors have compared their findings with those or other studies and discussed the possible applicability of their results.

In general, appropriate methods are used for the study. However, the study has limitations in estimating desirability, cost reports and lack of probabilistic sensitivity analysis. It appears that the authors have provided a correct discussion of their analysis.

*Chesson* *et al.,* *2008:*

The ICERs were presented in this study. The method of this study was mentioned online. The sensitivity analysis investigated the issue of uncertainty, using a deterministic approach, which was useful in terms of identifying the most influential model inputs. 

Based on the modeling of cost effectiveness, six studies have been selected and categorized as follows:

- Brisson *et al*., 2007, Kulasingam *et al.,* 2007, Bergeron *et al*., 2008, and Kulasingam *et al*., 2008 made use Morkov Models
- Elbasha *et al*., 2007, and Chesson *et al*., 2008 made use of Dynamic Models

A number of limitations are included in all discussed models. The studies, which used Markov models, did not take into account the herd immunity, which may result in underestimating the cost effectiveness of vaccination. The studies that used dynamic transmission models did not consider the homosexual and bisexual effect of vaccination, which is not very important in Iran. 

Among these six models and based on the available epidemiologic data in Iran, Chesson *et al.* 2008, is simplified and it requires substantially fewer assumptions than the other more complex Markov and hybrid models do. Therefore, we decided to use this model for the evaluation of cost effectiveness of Gardasil in Iran. On the other hand, this simplified model was compared to previous complicated Markov, hybrid and dynamic models like the Markov model of Goldie et al (45), the Markov model of Sanders and Taira (46), the hybrid model of Taira *et al.* (47), and the dynamic model of Elbasha *et al. *(25). The findings were consistent with those from other published cost-effectiveness models (48).

Another advantage of this model is that there is no need to model the probability of HPV acquisition, the possible progression from HPV infection to CIN I, CIN II, CIN III and cervical cancer, and the probability of HPV transmission, which are not available in Iranian epidemiologic data. Age-specific incidence rates of cervical cancer (ASIR CC) is available in Iran. It is mentioned in 2008 population-based cancer registries in Iran. This model needs the following data which are available in Iran:

- Age-specific incidence rates of cervical cancer
- Treatment cost of HPV adverse health outcomes
- Costs Averted by vaccination
- QALYs Saved by vaccination

## Conclusion

Cervical Cancer would be considered as a preventable cancer by vaccination. Generally HPV vaccine will have an influential impact on prevention of Cervical Cancer and finally on the epidemiology of HPV related Cancers. The most important note for using HPV vaccine is the age of individuals and their history of sexual activities. Most of the models compared the screening with vaccination and all included studies showed that adding this vaccine to the national vaccination program will be cost- effective based on the cost-effectiveness threshold of 50,000 USD per QALY. As most of these studies were done in the United States, mentioned cost per QALY is suitable for USA. For developing countries like Iran, World Health Organization (WHO) has recommended a cost-effectiveness threshold indicating that a healthcare technology is cost effective if the ICER is less than three times the GDP (Gross Domestic Production) per capita WHO’s recommendation about threshold of developing countries considers ICER less than triplet of GDP of Iran for 2012 is 5,810 $. Based on WHO recommendation, ICER less than 17,430 USD per QALYs could be considered cost-effective. The Chesson *et al.,* 2008 model is simple and could be applicable in different countries with limited data. On the other hand, the results of this model were consistent with published studies based on the more complex models whereas key assumptions have been similar. The authors stated and demonstrated that their findings were consistent with those from other published cost-effectiveness studies. The main advantage of this model was its simplicity, which required fewer assumptions compared with more complex models. The biggest drawback of their analysis, as the authors stated, was the limited understanding of the impact of changes in screening strategies on the cost-effectiveness of HPV vaccination.
